# Enhanced Proteolytic and Glycooxidative Activity in Visceral Adipose Tissue in Obesity: A Tissue-Level Comparative Study

**DOI:** 10.3390/ijms27125371

**Published:** 2026-06-14

**Authors:** Konrad Wiśniewski, Barbara Choromańska, Mateusz Maciejczyk, Alan Tkaczuk, Andrzej Kupisz, Roman Cemaga, Jacek Dadan, Małgorzata Żendzian-Piotrowska, Anna Zalewska, Piotr Andrzej Myśliwiec

**Affiliations:** 1 1st Department of General and Endocrine Surgery, Medical University of Bialystok, 24a M. Sklodowskiej-Curie Street, 15-276 Bialystok, Poland; barbara.choromanska@umb.edu.pl (B.C.); alan.tkaczuk@onet.eu (A.T.); jacdad@poczta.onet.pl (J.D.); piotr.a.mysliwiec@gmail.com (P.A.M.); 2 Department of Hygiene, Epidemiology and Ergonomics, Medical University of Bialystok, 2c A. Mickiewicza Street, 15-233 Bialystok, Poland; mat.maciejczyk@gmail.com (M.M.); mzpiotrowska@gmail.com (M.Ż.-P.); 3 Department of General Surgery, Humana Medica Omeda, 39 Fabryczna Street, 15-482 Bialystok, Poland; a.kupisz@omeda.pl; 4 Students’ Scientific Club “Biochemistry of Civilization Diseases” Department of Hygiene, Epidemiology and Ergonomics, Medical University of Bialystok, 2c A. Mickiewicza Street, 15-233 Bialystok, Poland; rcemaga@gmail.com; 5 Independent Laboratory of Experimental Dentistry, Medical University of Bialystok, 24a M. Sklodowskiej-Curie Street, 15-274 Bialystok, Poland; azalewska426@gmail.com

**Keywords:** obesity, visceral adipose tissue, subcutaneous adipose tissue, matrix metalloproteinases, MMP activity, extracellular matrix remodeling, advanced glycation end-products, glycooxidation, oxidative stress, plasma biomarkers

## Abstract

Adipose tissue expansion in obesity is accompanied by extracellular matrix (ECM) remodeling, regulated by matrix metalloproteinases (MMPs). Visceral adipose tissue (VAT) is metabolically more active than subcutaneous adipose tissue (SAT). However, depot-specific differences in proteolytic activity and protein glycooxidation remain incompletely characterized. In this case–control study, we assessed the activity of six matrix metalloproteinases (MMP-1, -2, -7, -9, -11, and -13) using a fluorescence resonance energy transfer (FRET) assay and quantified advanced glycation- and glycooxidation-related markers in paired VAT, SAT, and plasma samples obtained from 40 patients with obesity and 21 non-obese controls. The activities of all assessed MMPs were greater in patients with obesity than in the control group (*p* < 0.01 for all MMPs). Direct tissue-compartment comparisons showed that MMP activity and glycooxidation-related markers were most pronounced in VAT, with markedly higher values in obese individuals compared with controls. In VAT of obese individuals, median MMP activity was approximately 50–60% higher compared with controls. Amyloid cross-β-structure, vesperlysine, and pentosidine were significantly elevated in VAT in obesity, whereas plasma levels were markedly lower and showed limited group differences. No significant differences were observed between obese participants with and without metabolic syndrome. Obesity is associated with a depot-specific molecular profile characterized by enhanced proteolytic and glycooxidative activity predominantly within visceral adipose tissue. These findings highlight the importance of tissue-compartment-specific assessment in obesity.

## 1. Introduction

Obesity is a complex, chronic disorder characterized not only by excess adiposity but also by profound alterations in adipose tissue biology [1,2]. Accumulating evidence indicates that qualitative changes within adipose tissue—rather than total fat mass alone—play a key role in the development of metabolic and cardiovascular complications [3,4]. Among different fat depots, visceral adipose tissue (VAT) is consistently associated with a higher risk of insulin resistance, type 2 diabetes, non-alcoholic fatty liver disease, and cardiovascular disease compared with subcutaneous adipose tissue (SAT) [5,6,7,8,9]. This suggests that depot-specific molecular and structural differences may contribute to the heterogeneous metabolic impact of adipose tissue distribution [5,7,8].

Adipose tissue expansion in obesity is accompanied by extracellular matrix (ECM) remodeling, altered proteolytic activity, immune cell infiltration, and oxidative stress [4,10,11]. ECM turnover is tightly regulated and essential for maintaining tissue architecture and mechanical properties [10,11,12]. Dysregulated ECM remodeling may influence adipocyte expandability, local inflammation, and tissue stiffness [4,11,12]. Matrix metalloproteinases (MMPs) constitute a family of zinc-dependent endopeptidases that regulate ECM degradation and remodeling [13,14]. Several MMPs, including MMP-1, -2, -7, -9, -11, and -13, have been implicated in adipose tissue remodeling, angiogenesis, and inflammatory signaling [10,15,16].

The selected MMP panel was designed to capture multiple aspects of extracellular matrix remodeling. MMP-1 and MMP-13 are major collagenases; MMP-2 and MMP-9 are gelatinases involved in basement membrane remodeling; and MMP-7 participates in extracellular matrix processing and inflammatory signaling, whereas MMP-11 has been associated with adipose tissue remodeling and metabolic adaptation. Together, these enzymes represent complementary proteolytic pathways potentially involved in obesity-related adipose tissue dysfunction.

However, most human studies have focused on circulating concentrations of selected MMPs rather than direct assessment of their enzymatic activity within specific adipose depots [16,17,18]. Importantly, circulating levels may not accurately reflect local proteolytic processes within adipose tissue, where ECM remodeling occurs in a spatially and functionally heterogeneous manner. Moreover, available studies typically investigate individual MMPs or single tissue compartments, which limits the ability to capture coordinated proteolytic activity across different fat depots.

Therefore, a comprehensive, depot-specific evaluation of MMP activity directly within human adipose tissue remains limited. In particular, studies simultaneously assessing multiple MMP isoforms and comparing their enzymatic activity between visceral and subcutaneous adipose tissue within the same individuals are scarce. Addressing this gap may provide a more integrated understanding of tissue-specific remodeling processes and their potential contribution to metabolic dysfunction in obesity.

In parallel, obesity is associated with increased oxidative stress and enhanced non-enzymatic protein modification [19,20]. AGEs and related glycooxidative structures accumulate as a result of chronic exposure to reactive carbonyl and oxygen species [21,22]. These modifications may alter protein structure, cross-link extracellular matrix components, and influence cellular signaling pathways [21,23,24]. Among the various glycooxidation-related products, pentosidine and vesperlysine are well-characterized advanced glycation end-products (AGEs) that reflect long-term carbonyl stress and protein cross-linking. In contrast, amyloid cross-β structures represent conformational alterations and aggregation of modified proteins that may occur under conditions of sustained oxidative and glycooxidative stress. These markers were selected because they capture complementary aspects of protein damage, including AGE accumulation, protein cross-link formation, and structural remodeling, which may contribute to extracellular matrix dysfunction and adipose tissue pathology in obesity. Although circulating AGE levels and AGE–RAGE signaling in adipose tissue have been investigated in the context of obesity and diabetes, data on depot-specific accumulation of glycooxidative modifications within human adipose tissue remain limited and incompletely characterized [24,25,26,27]. In particular, direct comparative analyses of visceral and subcutaneous adipose tissue are scarce, which highlights an important gap in current knowledge [8,10,11].

Importantly, VAT and SAT differ in vascularization, inflammatory cell composition, endocrine activity, and metabolic responsiveness [6,7,8,9,28]. Nevertheless, although individual aspects of adipose tissue remodeling, proteolytic activity, and AGE–RAGE signaling have been investigated, comprehensive comparative analyses integrating proteolytic activity and glycooxidation-related protein modification across paired VAT, SAT, and plasma samples in individuals with and without obesity remain limited [10,16,25]. In particular, studies combining these pathways within the same experimental framework and directly comparing tissue compartments are scarce, highlighting a relevant gap in current knowledge [10,25].

A more detailed characterization of depot-specific molecular patterns may improve understanding of adipose tissue heterogeneity in obesity and clarify to what extent circulating biomarkers reflect tissue-level alterations [7,17,18]. Therefore, the aim of the present case–control study was to compare matrix metalloproteinase activity and glycooxidation-related markers in visceral and subcutaneous adipose tissue and plasma obtained from individuals with and without obesity.

## 2. Results

### 2.1. Study Population

The study included 40 patients with morbid obesity undergoing bariatric surgery and 21 non-obese control participants. Patients with obesity presented significantly higher body weight and body mass index (BMI) compared with controls (both *p* < 0.0001) [1,2]. Systolic and diastolic blood pressure were also significantly elevated in the obesity group (*p* = 0.0008 and *p* < 0.0001, respectively) [3]. White blood cell (WBC) count was higher in participants with obesity [3,28]. Hematological parameters such as hemoglobin and hematocrit were lower in the obesity group, whereas most electrolyte and coagulation parameters were comparable between groups.

Detailed anthropometric, biochemical, and hematological characteristics are presented in Table 1.

### 2.2. Matrix Metalloproteinase Activity

Direct comparisons between obese and control participants within each tissue compartment showed significantly higher activity of all analyzed MMPs in the VAT of obese individuals. In VAT, median MMP activity in obese participants was higher by 54.7% for MMP-1, 54.5% for MMP-2, 56.6% for MMP-7, 53.1% for MMP-9, 58.2% for MMP-11, and 57.6% for MMP-13 compared with controls. This consistent increase across all analyzed MMPs indicates enhanced proteolytic activity predominantly within VAT.

Complementary two-way ANOVA confirmed significant effects of tissue compartment and obesity status, as well as obesity × tissue interactions for all analyzed MMPs. These results are presented in Supplementary Table S4.

In SAT, obesity-related differences were smaller than in VAT and did not remain statistically significant after correction for multiple comparisons for any of the analyzed MMPs.

In plasma, absolute MMP activity was markedly lower than in adipose tissue compartments. Although median values tended to be higher in obese individuals, group differences were modest and did not remain statistically significant after correction for multiple comparisons for any of the analyzed MMPs.

**Figure 1:**
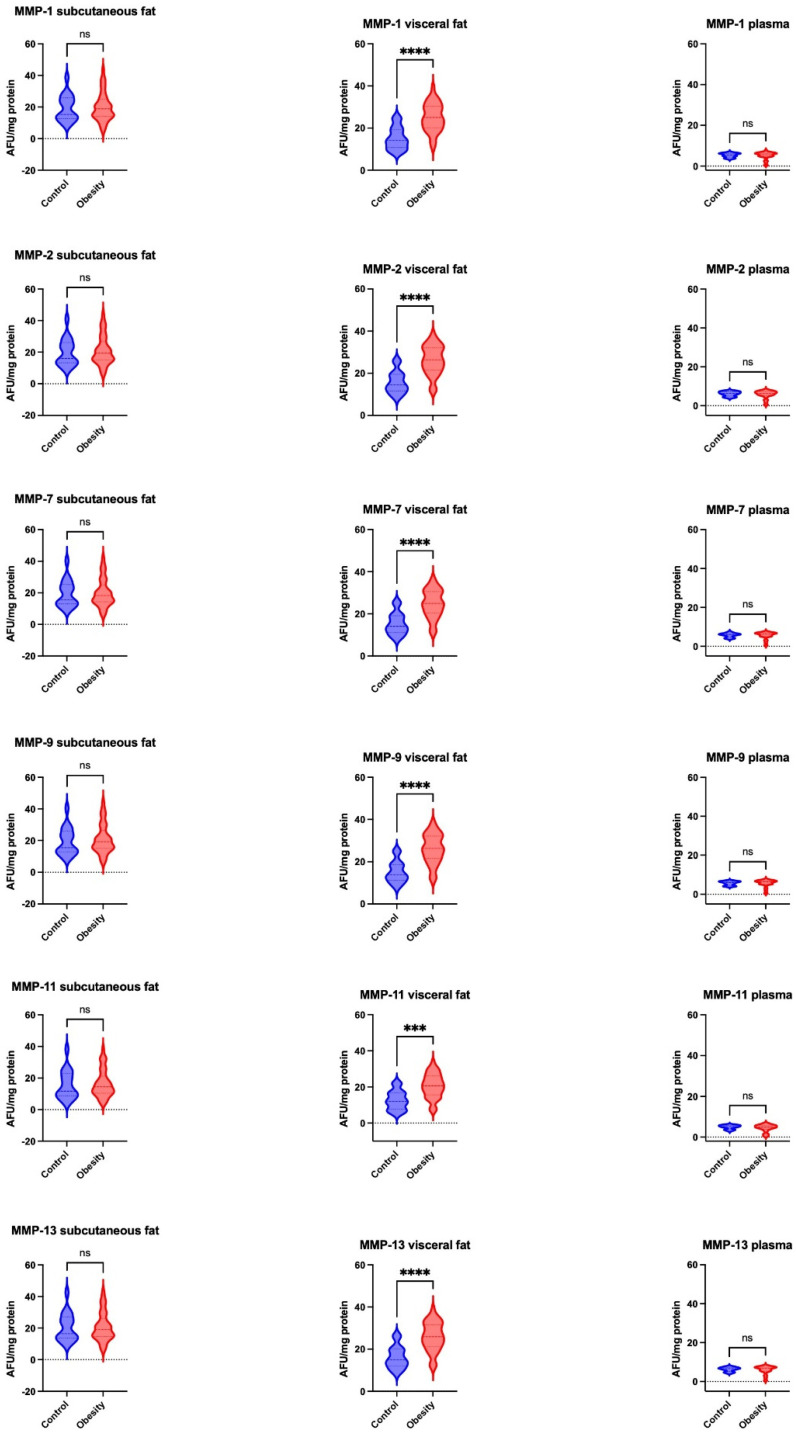
Pairwise comparison of matrix metalloproteinase activity (MMP-1, -2, -7, -9, -11, and -13) between obese and control participants within each tissue compartment (VAT, SAT, and plasma). Data are presented as violin plots with medians and interquartile ranges. MMP activity is expressed as arbitrary fluorescence units normalized to protein content (AFU/mg protein). Statistical significance was assessed using Mann–Whitney U tests. Asterisks indicate significance levels (*** *p* < 0.001, **** *p* < 0.0001; ns—not significant).

The distribution of MMP activity across tissue compartments and study groups is presented in Supplementary Figure S1. Direct pairwise comparisons within each tissue compartment are shown in Figure 1.

### 2.3. Protein Modification and Glycooxidation Markers

Direct comparisons within each tissue compartment showed that selected glycooxidation-related markers were significantly elevated in VAT of obese individuals compared with controls. In VAT, amyloid cross-β-structure levels were 61.9% higher, vesperlysine levels were 69.8% higher, and pentosidine levels were 72.8% higher in obese participants. These findings indicate that obesity-related glycooxidative protein modifications are most pronounced in visceral adipose tissue.

Differences in SAT and plasma were smaller and were not consistently significant. Dityrosine and total AGEs showed no significant obesity-related differences within individual compartments, although their levels differed between tissue compartments. Overall, glycooxidation-related markers demonstrated a pattern similar to MMP activity, with the most pronounced alterations observed in VAT.

Complementary two-way ANOVA confirmed a significant tissue-compartment effect for all analyzed fluorescence-based markers and significant obesity-related effects for selected markers. These results are presented in Supplementary Table S5.

**Figure 2:**
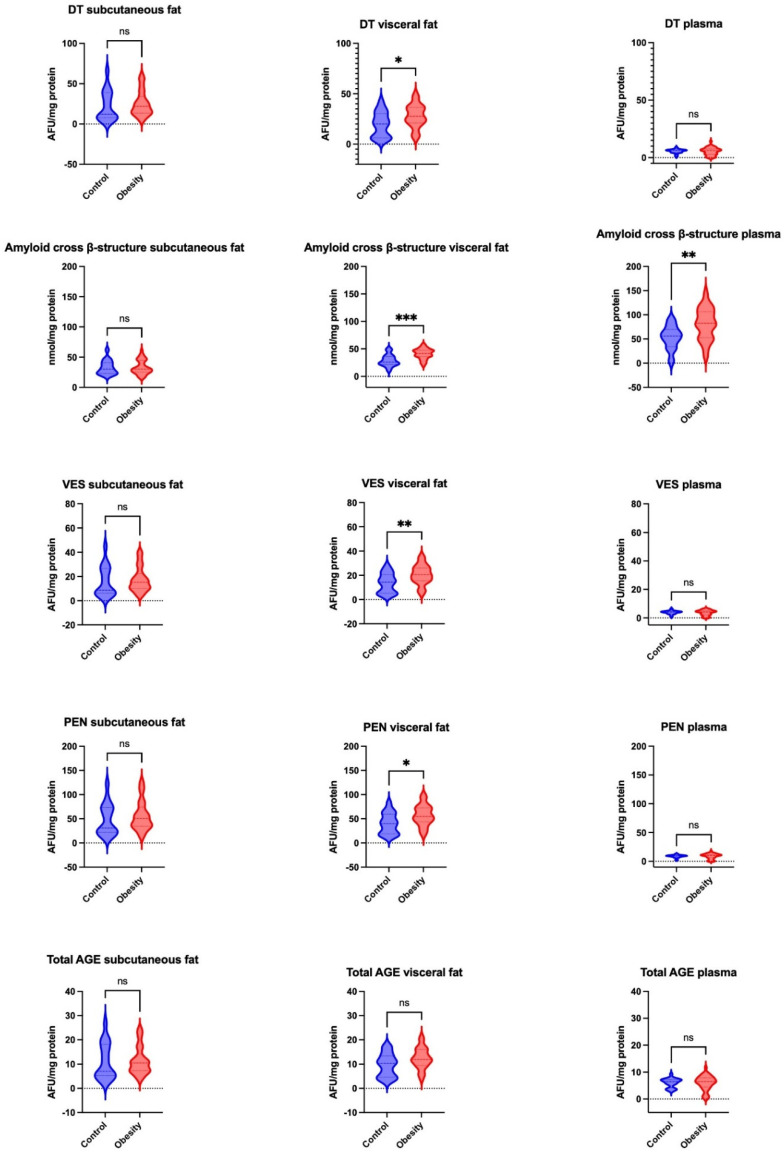
Pairwise comparison of AGE-related markers between obese and control participants within each tissue compartment (VAT, SAT, and plasma). Data are presented as violin plots with medians and interquartile ranges. Fluorescence intensity is expressed as arbitrary fluorescence units normalized to protein content (AFU/mg protein). Statistical significance was assessed using Mann–Whitney U tests. Asterisks indicate significance levels (* *p* < 0.05, ** *p* < 0.01, *** *p* < 0.001; ns—not significant).

For clarity, the overall distribution across tissue compartments is presented in Supplementary Figure S2, whereas direct obese-versus-control comparisons within individual tissue compartments are shown in Figure 2.

### 2.4. Comparison Between Obese Participants with and Without Metabolic Syndrome

Subgroup analysis within the obesity cohort revealed no statistically significant differences between individuals with and without metabolic syndrome for any analyzed MMPs or AGE-related markers in plasma, SAT, or VAT (all *p* > 0.30).

Median values and interquartile ranges were comparable between subgroups across all compartments. Detailed results are provided in Supplementary Tables S1–S3. The lack of significant differences between MetS subgroups was consistent across all tissue compartments and markers.

### 2.5. Correlation Analyses

Spearman correlation analyses were performed to explore associations between VAT molecular markers and selected anthropometric and laboratory parameters.

No significant correlations were observed between VAT MMP activity or AGE-related markers and BMI, body weight, or blood pressure (all *p* > 0.05). No consistent associations were detected with CRP or fibrinogen.

Moderate correlations were identified between selected VAT glycooxidation markers and serum amylase (r = 0.41–0.48; *p* < 0.05). Dityrosine and vesperlysine levels in VAT were inversely correlated with white blood cell count (r ≈ −0.37; *p* < 0.05).

MMP-11 activity in VAT correlated positively with serum amylase (r = 0.403; *p* = 0.046) and negatively with WBC (r = −0.382; *p* = 0.024). MMP-1 showed a modest inverse association with WBC (r = −0.356; *p* = 0.036).

Other correlations did not reach statistical significance.

## 3. Discussion

Obesity is associated with complex alterations in adipose tissue structure and function that extend beyond quantitative fat accumulation [1,2,4]. In the present case–control study, we demonstrate that both matrix metalloproteinase activity and glycooxidation-related protein modifications are markedly higher in adipose tissue than in plasma, with the most pronounced alterations consistently observed in VAT.

Direct tissue-compartment comparisons revealed a consistent pattern of increased MMP activity and selected glycooxidation-related markers predominantly in VAT of obese individuals. Complementary two-way ANOVA supported the presence of significant compartment-specific effects, indicating that obesity-related molecular alterations were not uniformly distributed across VAT, SAT, and plasma. Together, these findings indicate a consistent depot-specific pattern in obesity [5,7,8].

A consistent observation across all analyzed metalloproteinases was the approximately 50–60% increase in enzymatic activity in VAT of obese individuals compared with controls. This uniform pattern across multiple MMPs suggests coordinated alterations in proteolytic activity within visceral fat [10,16]. Previous experimental and clinical studies have implicated selected MMPs, particularly MMP-2 and MMP-9, in adipose tissue remodeling and cardiometabolic risk [17,29,30]. However, most available human data rely on circulating concentrations rather than direct assessment of enzymatic activity in adipose depots [16,18]. Our findings extend these observations by demonstrating that active proteolytic signaling is predominantly localized within VAT rather than being reflected in plasma [15].

Although increased MMP activity was consistently observed in VAT, the present study did not assess tissue inhibitors of metalloproteinases (TIMPs), which constitute an important regulatory component of extracellular matrix turnover. Therefore, the observed increase in proteolytic activity should be interpreted as enhanced proteolytic potential rather than direct evidence of altered MMP/TIMP balance. Future studies integrating both MMP activity and TIMP expression may provide a more comprehensive characterization of adipose tissue remodeling in obesity.

It should also be noted that the present study did not assess membrane-type matrix metalloproteinases, particularly MMP-14 (MT1-MMP), which has been recognized as an important regulator of adipose tissue extracellular matrix remodeling and collagen turnover. Previous studies have demonstrated a critical role of MMP-14 in adipose tissue expansion and structural adaptation during obesity. Therefore, although the current findings indicate enhanced proteolytic activity in VAT, they do not provide a complete characterization of all metalloproteinase pathways involved in adipose tissue remodeling. Future studies integrating both soluble and membrane-associated MMPs may offer a more comprehensive understanding of depot-specific ECM dynamics.

An additional methodological aspect that should be considered is the use of APMA pre-incubation to activate latent pro-MMPs prior to enzymatic activity assessment. Consequently, the obtained measurements reflect total potential MMP activity rather than endogenous in vivo activity present at the time of tissue collection. This approach was intentionally selected to evaluate the overall proteolytic capacity of the investigated tissue compartments and to enable standardized comparisons across samples. However, the results should not be interpreted as a direct measure of physiological MMP activity under native tissue conditions.

The markedly lower MMP activity observed in plasma, together with modest or absent group differences in circulation, supports the concept that circulating markers may incompletely represent tissue-level remodeling processes [17,18]. This discrepancy may be explained by several factors, including limited release of active enzymes from the tissue into the circulation, rapid inhibition by circulating tissue inhibitors of metalloproteinases (TIMPs), or temporal differences between local enzymatic activity and its systemic reflection [14]. In addition, dilution effects and the complex dynamics of protein clearance from plasma may further attenuate detectable differences at the systemic level [14].

Additional mechanisms may also contribute to the observed compartment-specific differences. Matrix metalloproteinases are subject to complex post-translational regulation, including intracellular trafficking, proteolytic processing, interaction with endogenous inhibitors, and degradation through the ubiquitin–proteasome system. Consequently, measured enzymatic activity may not directly reflect local protein abundance, and tissue and circulating levels may differ substantially due to differences in enzyme turnover, degradation, and clearance. These regulatory processes may further explain why pronounced alterations detected in VAT were only weakly reflected in plasma.

This compartmental discrepancy underscores the importance of direct tissue assessment when investigating alterations associated with obesity [4,10]. From a clinical perspective, this finding may help explain why circulating biomarkers often show limited sensitivity in reflecting early or localized adipose tissue dysfunction, despite the presence of metabolically relevant alterations within visceral fat.

In parallel, markers of protein glycation and glycooxidation were significantly higher in adipose tissue than in plasma. Among these markers, amyloid cross-β structure, vesperlysine, and pentosidine demonstrated significant obesity-related increases, particularly within VAT. The simultaneous increase in these markers suggests not only enhanced AGE accumulation but also increased protein cross-linking and structural modification within visceral adipose tissue. In contrast, dityrosine and total AGE exhibited strong tissue effects. These results indicate that glycooxidative modifications preferentially accumulate within adipose tissue rather than being reflected in circulation [23,25].

The observed increase in proteolytic activity and glycooxidative modifications within VAT may also have implications for ECM organization and mechanical properties [10,11,12]. Accumulation of AGEs can promote cross-linking of matrix proteins, potentially contributing to increased tissue stiffness and altered adipose tissue expandability [11,21,23]. Such structural changes have been linked to impaired adipocyte function and may limit the capacity of adipose tissue to store excess energy in a metabolically safe manner [4,11,12].

In this context, enhanced ECM remodeling and glycooxidative stress within VAT may also be indirectly related to the development of insulin resistance, as both processes have been associated with adipose tissue dysfunction and altered metabolic signaling, which are key features of obesity-related metabolic complications, including type 2 diabetes and cardiovascular disease [7,26,27,28]. Although the present study was not designed to assess insulin sensitivity directly, the observed molecular pattern is consistent with mechanisms previously implicated in metabolic dysregulation [9,25,26].

Furthermore, potential interactions between AGE accumulation and proteolytic activity should be considered [23,25]. Activation of the AGE–RAGE signaling pathway has been shown to promote inflammatory and oxidative responses, which may influence MMP expression and activity [14,24,25,26,27]. This suggests a possible link between glycooxidative stress and ECM remodeling; however, this relationship cannot be established based on the current data and warrants further investigation [10,23].

VAT is characterized by distinct vascularization, immune cell composition, endocrine activity, and metabolic responsiveness compared with SAT [6,7,8,28]. Numerous studies have demonstrated stronger inflammatory activation, higher lipolytic activity, and a closer association with cardiometabolic risk factors in visceral fat [5,6,7,9]. These biological characteristics may explain why obesity-related increases in MMP activity and glycooxidative protein modifications were predominantly observed in VAT, whereas corresponding alterations in SAT were less pronounced and did not remain statistically significant after correction for multiple comparisons. Together, these findings suggest that extracellular matrix remodeling and glycooxidative stress preferentially affect the metabolically more active visceral compartment during obesity.

Given the well-established association between visceral adiposity and cardiometabolic risk, these molecular alterations may represent tissue-level mechanisms contributing to obesity-related complications. However, the present findings remain descriptive and do not establish causal relationships between the observed molecular alterations and clinical outcomes.

Subgroup analysis revealed no significant differences in MMP activity or AGE-related markers between obese individuals with and without metabolic syndrome. This observation suggests that the VAT depot here is primarily associated with obesity itself rather than with the clinical diagnosis of metabolic syndrome [19,31,32]. Alternatively, the absence of subgroup differences may reflect limited statistical power. Because the study was not specifically designed or powered to detect differences between metabolic syndrome subgroups, small-to-moderate effects may have remained undetected. Therefore, the lack of statistically significant findings should not be interpreted as definitive evidence that metabolic syndrome has no influence on the analyzed molecular parameters. Larger studies incorporating detailed metabolic phenotyping would be required to clarify whether these molecular alterations relate to specific metabolic traits [7,33]. It is also possible that the molecular alterations observed in VAT represent early or fundamental changes associated with obesity itself, which are not further differentiated by the clinical classification of metabolic syndrome [4,33,34].

An important aspect of the present findings is the discrepancy between tissue-level alterations and circulating markers. Despite pronounced changes in VAT, plasma levels of MMP activity and glycooxidation-related markers showed only modest or non-significant differences between obese and control individuals. This suggests that circulating biomarkers may underestimate the extent of local adipose tissue remodeling. Clinically, this may contribute to the limited ability of standard blood-based markers to fully capture early or tissue-specific pathological processes associated with obesity. Therefore, the observed tissue-specific molecular alterations may precede detectable systemic changes and could be more directly linked to the development of metabolic complications.

Several limitations should be considered when interpreting these results. The case–control design precludes causal inference and does not allow for the assessment of temporal relationships between obesity and the observed molecular alterations. The sample size, particularly in subgroup analyses, may limit statistical power to detect small-to-moderate differences. In addition, although enzymatic activity and glycooxidative markers provide functional information, we did not directly assess extracellular matrix composition, tissue biomechanics, receptor signaling, or longitudinal metabolic outcomes [11,25,35]. Therefore, the biological consequences of the observed molecular pattern remain to be established.

Despite these limitations, the study provides a comprehensive comparative assessment of multiple MMPs and glycooxidation markers across paired visceral and subcutaneous adipose tissue samples and plasma. The consistent depot-specific pattern observed across independent molecular pathways strengthens the robustness of the findings.

In summary, obesity was associated with enhanced proteolytic and glycooxidative activity predominantly within visceral adipose tissue, whereas corresponding plasma alterations were considerably less pronounced. These findings emphasize the importance of tissue-compartment-specific assessment and suggest that circulating biomarkers may incompletely reflect local adipose tissue remodeling. Further studies integrating molecular profiling with structural and longitudinal clinical data are warranted to clarify the biological and clinical relevance of these compartment-specific differences.

## 4. Materials and Methods

### 4.1. Study Population and Clinical Data Collection

This case–control study included 40 patients with severe obesity who qualified for bariatric surgery according to institutional eligibility criteria. All participants had a body mass index (BMI) > 32 kg/m^2^. Exclusion criteria included active malignancy, acute inflammatory disease, autoimmune disorders, chronic infectious diseases, significant alcohol abuse, and active nicotine dependence.

The control group consisted of 21 non-obese individuals (BMI < 26 kg/m^2^) without clinically diagnosed metabolic disorders who were scheduled for elective non-metabolic surgical procedures (hernia repair or laparoscopic cholecystectomy). None of the control participants had a history of bariatric or metabolic surgery. Control participants had no documented history of diabetes, chronic inflammatory disease, or metabolic pharmacotherapy based on medical records.

Anthropometric parameters (body weight, height, and BMI) were recorded prior to surgery. Venous blood samples were obtained after an overnight fast for routine biochemical and hematological analyses, including CRP, fibrinogen, liver enzymes, renal function parameters, glucose, and complete blood count.

Metabolic syndrome was diagnosed according to the International Diabetes Federation (IDF) criteria, requiring central obesity plus at least two of the following: elevated triglycerides, reduced HDL cholesterol, elevated blood pressure or previously diagnosed hypertension, and impaired fasting glucose or type 2 diabetes [33].

### 4.2. Adipose Tissue and Plasma Sample Collection

Visceral adipose tissue (VAT) samples were obtained intraoperatively from the greater omentum during abdominal cavity access. Subcutaneous adipose tissue (SAT) samples were collected from the abdominal wall incision site at the beginning of the procedure.

All tissue samples were collected prior to any major tissue manipulation. Immediately after excision, samples were rinsed in sterile saline, snap-frozen in liquid nitrogen, and stored at −80 °C until further analysis.

Blood samples were centrifuged at 4 °C within 30 min of collection, and plasma was aliquoted and stored at −80 °C. All samples were processed under identical pre-analytical conditions.

Laboratory analyses were performed in technical duplicates. Investigators performing biochemical measurements were blinded to clinical group allocation.

### 4.3. Ethical Approval and Informed Consent

The study was conducted in accordance with the Declaration of Helsinki and approved by the Bioethics Committee of the Medical University of Bialystok (resolution No. APK.002.98.2023 of 16 February 2023). Additional approval was granted by resolution No. R-I-002/475/2019 of 19 September 2024, authorizing the Humana Medica Omeda center as an additional site for biological sample collection and permitting continuation of the medical experiment at this location. Written informed consent was obtained from all participants prior to inclusion in the study.

### 4.4. Assessment of Matrix Metalloproteinase Activity

**Figure 3:**
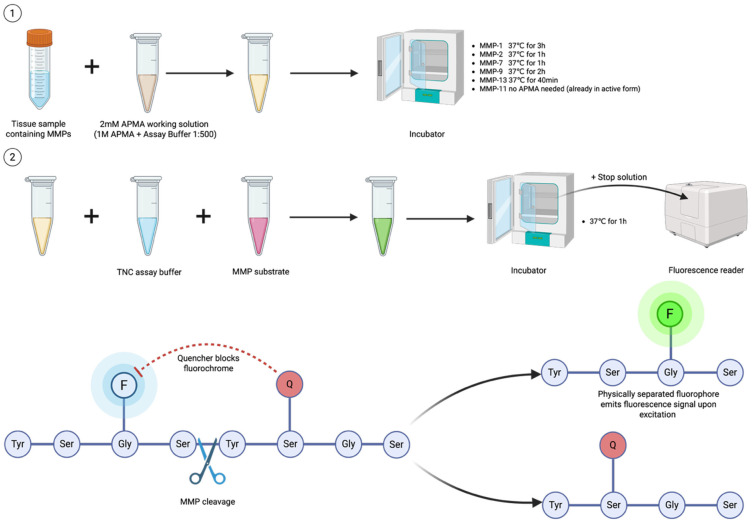
Schematic representation of the fluorescence resonance energy transfer (FRET)-based assay used to quantify matrix metalloproteinase (MMP) activity in adipose tissue and plasma samples. APMA—4-Aminophenylmercuric acetate; MMPs—metalloproteinases; TNC—Tris-HCl/NaCl/CaCl_2_. Created in BioRender. Maciejczyk, M. (2025) https://BioRender.com/qhulq83.

Matrix metalloproteinase (MMP) activity in VAT, SAT, and plasma was assessed using a fluorescence resonance energy transfer (FRET)-based assay as previously described [36]. A schematic representation of the process is shown in Figure 3.

Tissue samples were homogenized in ice-cold buffer and centrifuged to obtain supernatants. Total protein concentration was determined using the Pierce BCA Protein Assay Kit (Thermo Fisher Scientific, Rockford, IL, USA), and MMP activity was normalized to total protein content [37].

Prior to the assay, samples were pre-incubated at 37 °C with 2 mM 4-aminophenylmercuric acetate (APMA) to activate latent pro-MMPs and assess total potential enzymatic activity. Incubation times were isoform-specific (3 h for MMP-1; 1 h for MMP-2 and MMP-7; 2 h for MMP-9; 40 min for MMP-13). MMP-11 did not require pre-incubation due to its predominantly active form (① in Figure 3).

After activation, samples were incubated with Tris-HCl/NaCl/CaCl_2_ assay buffer and 3 µM fluorogenic substrate (MCA-Pro-Leu-Gly∼Leu-Dpa(Dnp)-Ala-Arg-NH_2_; Sigma-Aldrich, St. Louis, MO, USA). Cleavage of the substrate by active MMPs resulted in fluorescence emission proportional to enzymatic activity. Fluorescence was measured after 1 h incubation at 37 °C (② in Figure 3).

All measurements were performed in duplicate under identical instrumental settings. Background fluorescence was subtracted for each sample. Inter-assay coefficient of variation did not exceed 10%.

### 4.5. Determination of Advanced Glycation End-Products and Glycooxidation Markers

Total advanced glycation end-products (AGEs) and specific AGE-related structures (vesperlysine, pentosidine, and amyloid cross-β-structure) were quantified fluorimetrically [20,21]. Pentosidine and vesperlysine were selected as representative fluorescent AGE cross-links formed during chronic glycation and glycooxidation processes, whereas amyloid cross-β structures were assessed as markers of protein conformational alterations and aggregation. In addition, dityrosine (DT), a marker of oxidative protein modification, was measured [20].

Samples were diluted in 0.1 M H_2_SO_4_ and analyzed using an Infinite M200 PRO multi-mode reader (Tecan Group Ltd., Männedorf, Switzerland). Excitation/emission wavelength pairs were as follows:

350/440 nm for total AGEs;

350/405 nm for vesperlysine;

335/385 nm for pentosidine;

365/480 nm for dityrosine;

435/485 nm for amyloid cross-β-structure.

Fluorescence intensity was standardized to quinine sulfate solution (0.1 mg/mL in 0.1 M H_2_SO_4_). All measurements were performed in duplicate and normalized to total protein content [37].

Background fluorescence was subtracted for each sample, and all measurements were conducted using identical instrument parameters to minimize inter-assay variability.

### 4.6. Statistical Analysis

Statistical analyses were performed using GraphPad Prism 9.0 (GraphPad Software, San Diego, CA, USA).

Distribution of continuous variables was assessed using the Shapiro–Wilk test. Because several variables deviated from normality, data are presented as medians, interquartile ranges, and full ranges. Violin plots illustrate distribution together with medians and interquartile ranges.

Group comparisons between obese and control participants within each tissue compartment were performed using the Mann–Whitney U test. This analysis was used as the primary approach for direct between-group comparisons in VAT, SAT, and plasma.

As a complementary analysis, two-way analysis of variance (ANOVA) followed by Sidak’s multiple-comparison test was used to evaluate the effects of obesity status, tissue compartment, and their interaction on biochemical parameters. The results of this complementary analysis are presented in the Supplementary Materials. Normality of residuals was assessed prior to ANOVA, and homogeneity of variances was verified using Levene’s test.

Correlations between biochemical variables and anthropometric or laboratory parameters were analyzed using Spearman’s rank correlation coefficients.

Given the exploratory nature of correlation analyses and the number of comparisons performed, no formal correction for multiple testing was applied to correlation results; findings were interpreted cautiously.

No a priori sample size calculation was performed due to the exploratory nature of the study. Statistical significance was set at *p* < 0.05.

## 5. Limitations

This study has several limitations that should be acknowledged when interpreting the findings.

First, the cross-sectional design precludes any inference regarding causality or temporal relationships between obesity and the observed molecular alterations. The results describe associations and compartment-specific patterns but do not establish mechanistic pathways.

Second, the sample size was moderate, particularly in subgroup analyses comparing obese individuals with and without metabolic syndrome. Because no a priori sample size calculation was performed for these exploratory subgroup comparisons, the study may have been underpowered to detect small-to-moderate differences. Consequently, the absence of statistically significant findings should be interpreted cautiously and does not necessarily indicate biological equivalence between the analyzed subgroups.

Third, although we assessed the enzymatic activity of multiple MMPs and quantified several glycooxidation markers, we did not directly evaluate extracellular matrix composition, collagen cross-linking, tissue stiffness, receptor signaling (e.g., RAGE activation), inflammatory cytokine profiles, or tissue inhibitors of metalloproteinases (TIMPs), which are important regulators of MMP activity. Therefore, the functional and structural consequences of the observed molecular alterations, as well as the balance between MMPs and their endogenous inhibitors, cannot be determined from the present dataset.

Additionally, we did not assess membrane-type metalloproteinases such as MMP-14 (MT1-MMP), which represent important regulators of extracellular matrix turnover and adipose tissue remodeling. Consequently, the study does not allow a comprehensive evaluation of the regulatory networks controlling adipose tissue remodeling and primarily reflects the activity of the selected soluble MMP isoforms included in the study design.

Furthermore, MMP activity was assessed following APMA-mediated activation of latent pro-MMPs. Therefore, the obtained measurements reflect total potential proteolytic activity rather than endogenous enzymatic activity under physiological in vivo conditions. While this approach allows standardized assessment of proteolytic capacity across tissue compartments, it may not fully capture the complex regulation of MMP activity occurring within native tissue microenvironments.

Furthermore, we did not evaluate molecular mechanisms regulating MMP turnover, including ubiquitin–proteasome-mediated degradation or intracellular processing pathways, which may contribute to compartment-specific differences in enzymatic activity.

Fourth, fluorescence-based detection of AGE-related structures provides semi-quantitative information and may be influenced by intrinsic tissue autofluorescence or spectral overlap [20,21]. Although background subtraction and standardized instrumental settings were applied to minimize analytical variability, these methodological characteristics should be considered when interpreting the results.

Fifth, the control group consisted of individuals undergoing elective surgery rather than metabolically characterized healthy volunteers from the general population, which may limit generalizability. Additionally, residual confounding related to diet, medication use, physical activity, or other unmeasured factors cannot be excluded.

Finally, no a priori sample size calculation was performed, and the study was exploratory in nature. Replication in larger, independent cohorts and longitudinal designs would be necessary to confirm the obtained results.

## 6. Conclusions

In this case–control study, we demonstrate that obesity is associated with a distinct depot-specific molecular profile characterized by enhanced matrix metalloproteinase activity and increased glycooxidative protein modification predominantly within visceral adipose tissue. These alterations were consistently more pronounced in visceral fat than in subcutaneous adipose tissue and were only partially reflected in plasma.

A major strength of the study is the simultaneous assessment of multiple MMP isoforms and glycooxidation-related markers in paired visceral and subcutaneous adipose tissue samples, together with plasma obtained from the same individuals. This integrated tissue-compartment approach revealed that obesity-related molecular alterations are concentrated within visceral adipose tissue and may not be adequately captured by circulating biomarkers alone.

These findings highlight the biological heterogeneity of adipose tissue depots and support the concept that visceral adipose tissue represents a key site of extracellular matrix remodeling and glycooxidative stress in obesity. Further studies integrating molecular profiling with structural, functional, and longitudinal clinical data are warranted to clarify the biological and clinical implications of these compartment-specific differences.

## Figures and Tables

**Table 1:** Baseline anthropometric, hematological, and biochemical characteristics of control and obese participants. Data are presented as medians (minimum–maximum). BMI—body mass index; INR—international normalized ratio; MCH—mean corpuscular hemoglobin; MCHC—mean corpuscular hemoglobin concentration; MCV—mean corpuscular volume; RBCs—red blood cells; WBCs—white blood cells.

Parameter	Control (Median [Min–Max])	Obesity (Median [Min–Max])	*p*-Value
Body weight (kg)	80.0 (69.0–87.0)	113.0 (82.0–185.0)	<0.0001
BMI (kg/m^2^)	24.51 (20.05–25.96)	39.24 (32.37–51.79)	<0.0001
Creatinine (mg/dL)	0.90 (0.68–1.06)	0.74 (0.52–1.24)	0.0783
Potassium (mmol/L)	4.3 (4.0–4.8)	4.3 (3.7–5.2)	0.7628
Sodium (mmol/L)	140 (137–143)	139 (136–146)	0.0612
WBCs (×10^3^/µL)	5.67 (4.38–8.40)	11.19 (5.73–17.92)	<0.0001
RBCs (×10^6^/µL)	4.72 (4.15–6.10)	4.43 (3.52–5.55)	0.0016
Hemoglobin (g/dL)	14.6 (12.6–16.1)	13.1 (10.4–14.9)	<0.0001
Hematocrit (%)	43.1 (37.3–47.3)	39.2 (31.6–46.1)	0.0009
MCV (fL)	88.5 (85.0–92.0)	88.4 (82.0–93.4)	0.8427
MCH (pg)	31.2 (30.7–31.7)	29.4 (25.3–32.6)	0.1159
MCHC (g/dL)	34.5 (25.2–47.3)	33.4 (30.0–35.9)	0.0094
Platelets (×10^3^/µL)	261 (87–419)	243 (123–353)	0.8991
INR	1.09 (0.99–1.27)	1.05 (0.93–1.41)	0.3611
Systolic blood pressure (mmHg)	127 (106–142)	141 (100–168)	0.0008
Diastolic blood pressure (mmHg)	76 (65–88)	95 (78–109)	<0.0001

## Data Availability

The data that support the findings of this study are available from the corresponding author upon reasonable request. Data are not publicly available due to personal data protection regulations.

## Supplementary Materials

The following supporting information can be downloaded at: https://www.mdpi.com/article/10.3390/ijms27125371/s1.

## Abbreviations

AFUs	arbitrary fluorescence units
AGEs	advanced glycation end-products
APMA	4-aminophenylmercuric acetate
BMI	body mass index
CRP	C-reactive protein
DT	dityrosine
ECM	extracellular matrix
FRET	fluorescence resonance energy transfer
HDL	high-density lipoprotein
IDF	International Diabetes Federation
INR	international normalized ratio
MCH	mean corpuscular hemoglobin
MCHC	mean corpuscular hemoglobin concentration
MCV	mean corpuscular volume
MetS	metabolic syndrome
MMPs	matrix metalloproteinases
RAGE	receptor for advanced glycation end-products
RBCs	red blood cells
SAT	subcutaneous adipose tissue
TIMPs	tissue inhibitors of metalloproteinases
VAT	visceral adipose tissue
WBCs	white blood cells
