# Supplementary material for: Enhanced Proteolytic and Glycooxidative Activity in Visceral Adipose Tissue in Obesity: A Tissue-Level Comparative Study
Source: Int J Mol Sci. 2026 Jun 14;27(12):5371. doi: 10.3390/ijms27125371 (PMC13299355; doi:10.3390/ijms27125371)
Supplement: Supplementary file 1 [file ijms-27-05371-s001.zip › ijms-4326674-supplementary.pdf]

Parameter	MetS-					MetS+					P-value
	Minimum	25th percentile	Median	75th percentile	Maximum	Minimum	25th percentile	Median	75th percentile	Maximum	
**MMP-1 (AFU/mg protein)**	0.43	4.60	5.72	6.29	7.76	0.35	4.87	5.61	6.65	7.00	0.658
**MMP-2 (AFU/mg protein)**	0.55	5.05	6.27	7.20	8.40	0.42	5.52	6.40	7.34	7.89	0.898
**MMP-7 (AFU/mg protein)**	0.46	4.93	6.32	6.86	7.55	0.45	5.11	6.09	6.89	7.61	0.787
**MMP-9 (AFU/mg protein)**	0.51	5.11	6.30	6.83	7.93	0.41	5.34	6.34	6.86	7.46	0.831
**MMP-11 (AFU/mg protein)**	0.56	3.66	4.89	5.75	6.93	0.31	4.61	4.88	5.87	6.41	0.809
**MMP-13 (AFU/mg protein)**	0.55	5.15	6.92	7.47	8.01	0.45	5.34	6.45	7.34	8.34	0.700
**DT (AFU/mg protein)**	0.14	2.53	5.81	7.49	14.11	0.06	3.39	6.24	7.72	9.49	0.787
**Amyloid cross β-structure (nmol/mg protein)**	30.87	53.13	80.59	116.30	147.50	11.93	43.37	84.61	97.28	116.60	0.391
**VES (AFU/mg protein)**	0.12	1.81	4.07	5.04	6.44	0.08	2.54	4.11	4.88	6.40	0.809
**PEN (AFU/mg protein)**	0.15	4.40	10.12	11.77	17.70	0.09	6.72	10.89	12.53	14.27	0.618
**Total AGE (AFU/mg protein)**	0.58	4.67	6.72	8.02	11.72	0.32	4.03	5.72	7.56	9.50	0.310

Table S1. Comparison of matrix metalloproteinase activity and advanced glycation end-products concentration in **plasma** between obese patients with and without metabolic syndrome. **AGE** – advanced glycation end-product; **DT** – dityrosine; **MetS–** - patients without metabolic syndrome; **MetS+** - patients with metabolic syndrome; **MMP** – matrix metalloproteinase; **PEN** – pentosidine; **VES** – vesperlysine

Parameter	MetS-					MetS+					P-value
	Minimum	25th percentile	Median	75th percentile	Maximum	Minimum	25th percentile	Median	75th percentile	Maximum	
**MMP-1 (AFU/mg protein)**	6.50	14.47	20.08	23.98	37.48	5.18	13.98	16.29	26.22	43.37	0.825
**MMP-2 (AFU/mg protein)**	7.35	15.30	20.20	26.00	37.74	6.07	15.14	17.32	27.22	43.83	0.934
**MMP-7 (AFU/mg protein)**	6.91	14.58	19.28	24.66	36.31	6.25	14.15	16.54	25.45	42.10	0.978
**MMP-9 (AFU/mg protein)**	7.25	15.31	20.24	25.89	38.12	6.56	14.86	17.36	26.72	44.20	0.978
**MMP-11 (AFU/mg protein)**	4.26	10.32	15.07	21.59	32.99	4.64	10.32	12.50	21.50	38.17	0.912
**MMP-13 (AFU/mg protein)**	7.14	15.10	20.10	25.98	37.29	6.27	14.51	17.26	26.40	43.07	>0.9999
**DT (AFU/mg protein)**	4.88	13.44	21.79	34.04	57.89	6.07	13.65	23.88	35.46	60.98	0.659
**Amyloid cross β-structure (nmol/mg protein)**	15.85	24.85	30.76	42.49	54.63	16.68	25.31	29.53	44.99	61.10	>0.9999
**VES (AFU/mg protein)**	3.98	10.21	15.21	22.99	40.21	5.00	10.20	16.70	22.87	40.40	0.679
**PEN (AFU/mg protein)**	13.54	33.81	52.38	73.74	113.40	14.73	35.56	49.01	77.08	125.90	0.720
**Total AGE (AFU/mg protein)**	3.64	7.32	10.43	14.46	22.56	4.82	7.23	10.79	14.67	24.33	0.741

Table S2. Comparison of matrix metalloproteinase activity and advanced glycation products concentration in **subcutaneous fat** between obese patients with and without metabolic syndrome. **AGE** – advanced glycation end-product; **DT** – dityrosine; **MetS–** - patients without metabolic syndrome; **MetS+** - patients with metabolic syndrome; **MMP** – matrix metalloproteinase; **PEN** – pentosidine; **VES** – vesperlysine

Parameter	MetS-					MetS+					P-value
	Minimum	25th percentile	Median	75th percentile	Maximum	Minimum	25th percentile	Median	75th percentile	Maximum	
**MMP-1 (AFU/mg protein)**	12.99	19.72	24.51	30.53	39.27	10.77	20.32	25.04	29.18	32.09	0.766
**MMP-2 (AFU/mg protein)**	12.68	20.86	25.53	32.17	38.58	11.43	21.84	26.53	30.77	35.59	0.913
**MMP-7 (AFU/mg protein)**	12.02	19.37	24.64	30.68	36.81	10.65	21.05	24.96	28.39	33.45	0.863
**MMP-9 (AFU/mg protein)**	12.61	20.34	25.87	32.21	38.65	11.18	22.10	26.21	29.81	35.12	0.863
**MMP-11 (AFU/mg protein)**	7.48	15.20	20.08	26.14	33.59	7.52	16.32	20.99	26.72	30.06	0.742
**MMP-13 (AFU/mg protein)**	12.69	20.54	25.48	31.63	39.03	11.40	21.99	26.15	30.62	35.20	0.913
**DT (AFU/mg protein)**	7.38	21.92	29.10	37.09	50.72	4.94	18.73	26.48	35.02	39.14	0.604
**Amyloid cross β-structure (nmol/mg protein)**	22.87	36.02	40.44	47.74	56.44	19.42	34.04	42.74	47.39	58.50	0.839
**VES (AFU/mg protein)**	5.93	15.84	20.78	28.40	36.86	4.14	14.31	20.60	24.54	27.90	0.520
**PEN (AFU/mg protein)**	20.61	45.32	57.14	72.69	100.00	14.97	38.69	54.53	71.13	78.87	0.604
**Total AGE (AFU/mg protein)**	4.89	9.76	12.11	16.56	21.57	4.49	9.11	12.00	14.51	16.22	0.404

Table S3. Comparison of matrix metalloproteinase activity and advanced glycation end-products concentration in **visceral fat** between obese patients with and without metabolic syndrome. **AGE** – advanced glycation end-product; **DT** – dityrosine; **MetS–** - patients without metabolic syndrome; **MetS+** - patients with metabolic syndrome; **MMP** – matrix metalloproteinase; **PEN** – pentosidine; **VES** – vesperlysine

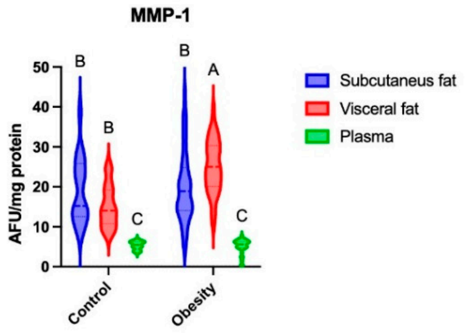

Source of Variation	% of total variation	P value	P value summary
Interaction	4.291	0.0001	***
Obesity	2.528	0.0011	**
Tissue	42.08	<0.0001	****

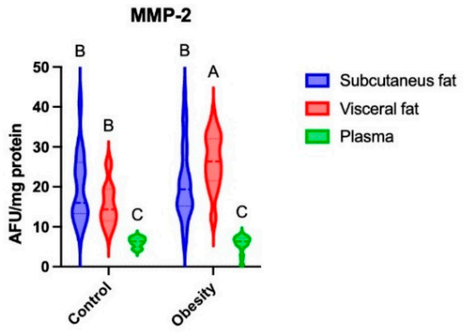

Source of Variation	% of total variation	P value	P value summary
Interaction	4.620	<0.0001	****
Obesity	2.926	0.0005	***
Tissue	40.67	<0.0001	****

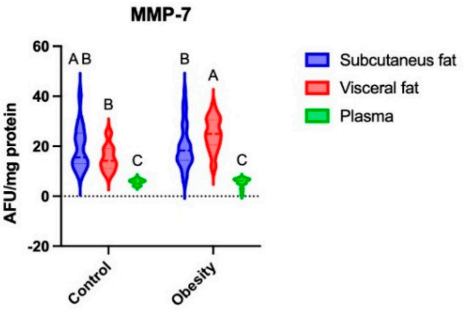

Source of Variation	% of total variation	P value	P value summary
Interaction	4.123	0.0003	***
Obesity	2.319	0.0021	**
Tissue	41.76	<0.0001	****

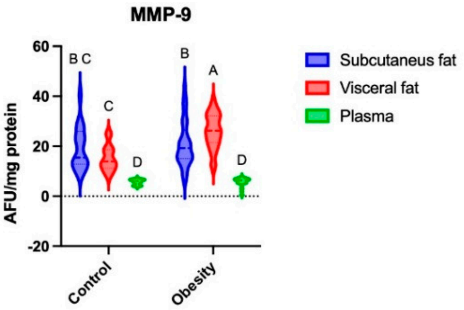

Source of Variation	% of total variation	P value	P value summary
Interaction	4.570	<0.0001	****
Obesity	3.341	0.0002	***
Tissue	40.43	<0.0001	****

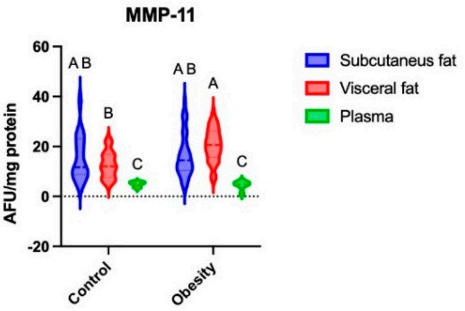

Source of Variation	% of total variation	P value	P value summary
Interaction	3.543	0.0032	**
Obesity	2.030	0.0098	**
Tissue	34.37	<0.0001	****

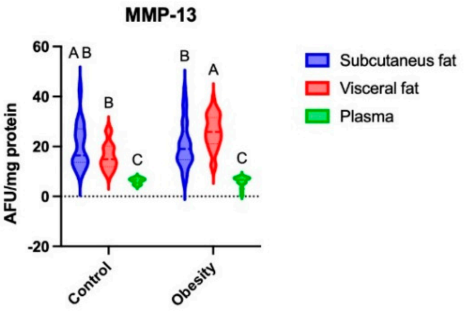

Source of Variation	% of total variation	P value	P value summary
Interaction	4.381	0.0002	***
Obesity	2.158	0.0030	**
Tissue	41.50	<0.0001	****

Figure S1. Matrix metalloproteinase activity (MMP-1, -2, -7, -9, -11 and -13) in visceral adipose tissue (VAT), subcutaneous adipose tissue (SAT) and plasma in obese and control participants. Data are presented as violin plots with median and interquartile range. MMP activity is expressed as arbitrary fluorescence units normalized to protein content (AFU/mg protein). Two-way ANOVA was used to assess the effects of obesity status, tissue type and their interaction. Within each obesity group, different letters indicate statistically significant differences between tissue compartments according to Sidak's multiple-comparison test ($p < 0.05$).

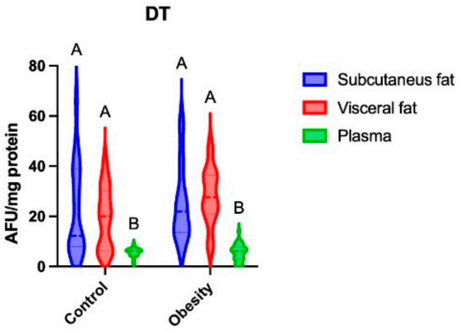

Source of Variation	% of total variation	P value	P value summary
Interaction	1.215	0.1911	ns
Obesity	1.234	0.0671	ns
Tissue	30.01	<0.0001	****

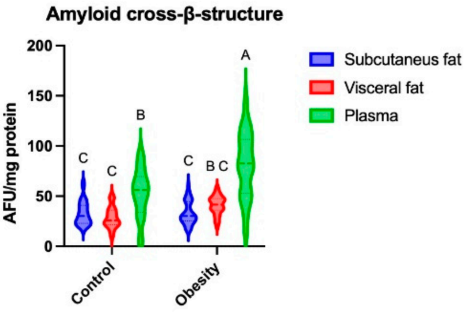

Source of Variation	% of total variation	P value	P value summary
Interaction	3.864	0.0025	**
Obesity	5.282	<0.0001	****
Tissue	29.15	<0.0001	****

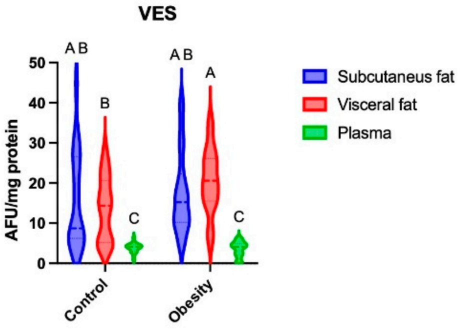

Source of Variation	% of total variation	P value	P value summary
Interaction	1.847	0.0661	ns
Obesity	1.515	0.0347	*
Tissue	32.39	<0.0001	****

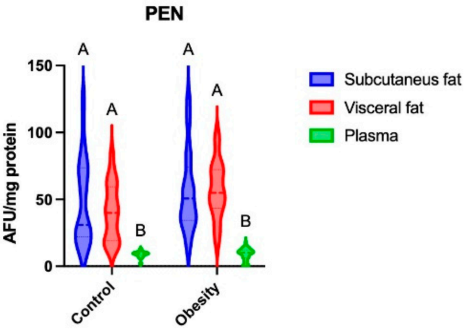

Source of Variation	% of total variation	P value	P value summary
Interaction	1.076	0.1720	ns
Obesity	1.522	0.0262	*
Tissue	37.90	<0.0001	****

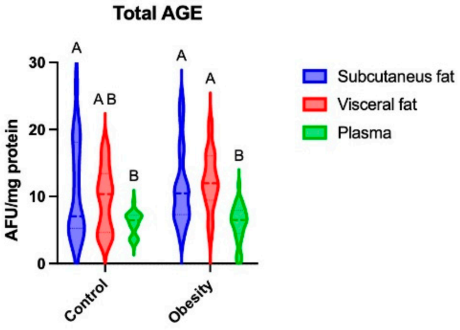

Source of Variation	% of total variation	P value	P value summary
Interaction	1.278	0.2477	ns
Obesity	0.8413	0.1753	ns
Tissue	18.67	<0.0001	****

Figure S2. Markers of protein glycation and glycooxidation (dityrosine, vesperlysine, pentosidine, amyloid cross-β-structure and total AGEs) in visceral adipose tissue (VAT), subcutaneous adipose tissue (SAT) and plasma in obese and control participants. Data are presented as violin plots with median and interquartile range. Two-way ANOVA was used to assess the effects of obesity status, tissue type and their interaction, followed by Sidak's multiple-comparison test. Within each obesity group, different letters indicate statistically significant differences between tissue compartments ($p < 0.05$).

Table S4 Complementary two-way ANOVA results for matrix metalloproteinase activity.

Marker	Obsesity effect p-value	Tissue compartment effect p-value	Obesity x tissue interaction p-value
MMP-1	0.0011	<0.0001	<0.01
MMP-2	0.0005	<0.0001	<0.01
MMP-7	0.0021	<0.0001	<0.01
MMP-9	0.0002	<0.0001	<0.01
MMP-11	0.0098	<0.0001	<0.01
MMP-13	0.0030	<0.0001	<0.01

Table S5 Complementary two-way ANOVA results for glycation and glycooxidation-related markers

Marker	Obsesity effect p-value	Tissue compartment effect p-value	Obesity x tissue interaction p-value
Dityrosine	0.0671	<0.0001	ns
Vesperlysine	0.0347	<0.0001	ns
Pentosidine	0.0262	<0.0001	ns
Amyloid cross-β structure	<0.0001	<0.0001	0.0025
Total AGEs	0.1753	<0.0001	ns
